# Supplementary material for: Content-rich biological network constructed by mining PubMed abstracts
Source: BMC Bioinformatics. 2004 Oct 8;5:147. doi: 10.1186/1471-2105-5-147 (PMC528731; doi:10.1186/1471-2105-5-147)
Supplement: Additional File 2 — The original results of the above study (non-essential files are deleted to keep the file size under the limit set by BMC bioinformatics). [file 1471-2105-5-147-S2.bz2 › chilibotAdditionalFile2/dip40/592ID9262406E10965/html/ARHGAP1_CDC42.html]

 


 **ARHGAP1** and **CDC42** 
  
Found 46 abstracts in PubMed, retrieved 40.  
 

 What does Google say? 
 PDF only 
| .edu only 

---

**Interactive relationship** (e.g. stimulation, inhibition, etc)

**Stimulatory relationship**- The Rho related small GTP binding protein  **Cdc42**  has a low intrinsic GTPase activity that is significantly enhanced by its specific GTPase activating protein, Cdc42GAP  [ **ARHGAP1** ] .  Ref: 9846874 Nat Struct Biol, 1998
- The RhoGAP  [ **ARHGAP1** ]  domain of Gmip stimulates in vitro the GTPase activity of RhoA, but is inactive towards other Rho family proteins such as Rac1 and  **Cdc42** .  Ref: 12093360 Biochem J, 2002
- The intrinsic rate of GTP hydrolysis by RhoA is relatively slow when compared to other Rho family GTPases such as  **Cdc42**  or Rac1 with a rate constant of 0.022 min 1, which can be further stimulated at least 4000 fold by p190 or p50RhoGAP  [ **ARHGAP1** ] .  Ref: 9548756 Biochemistry, 1998
**Neutral relationship**- The GTPase activity of TC10 was lower than that of  **Cdc42** , and TC10 possessed a lower affinity for, but greater responsiveness to, the p50Rho GTPase activating protein p50RhoGAP  [ **ARHGAP1** ]  than did  **Cdc42** .  Ref: 9799731 Curr Biol, 1998
- Structures of  **Cdc42**  bound to the active and catalytically compromised forms of Cdc42GAP  [ **ARHGAP1** ] .  Ref: 9846874 Nat Struct Biol, 1998
- The Cdc42GAP  [ **ARHGAP1** ]  stabilizes both the switch I and switch II domains of  **Cdc42**  and contributes a highly conserved arginine Arg 305 to the active site.  Ref: 9846874 Nat Struct Biol, 1998
- Together, these findings indicate that during cytokinesis, MgcRacGAP, previously known as a GAP for Rac  **Cdc42** , is functionally converted to a RhoGAP  [ **ARHGAP1** ]  through phosphorylation by Aurora B.  Ref: 12689593 Dev Cell, 2003
- These quantitative analysis provide insight that Cdc42GAP  [ **ARHGAP1** ]  functions as an effective negative regulator of  **Cdc42**  by fast, relatively tight binding to the GTP bound  **Cdc42** .  Ref: 9268338 J Biol Chem, 1997
- Arg 85 of rhoGAP  [ **ARHGAP1** ]  interacts with the P loop of Cdc42Hs  [ **CDC42** ] , but from biochemical data and by analogy with the G protein subunit G i alpha1, we propose that it adopts a different conformation during the catalytic cycle which enables it to stabilize the transition state of the GTP hydrolysis reaction.  Ref: 9262406 Nature, 1997
- The catalytic efficiencies Kcat Km of the GAP domains of Bcr, 3BP 1, and p190 on  **Cdc42**  are found to be 6 60, and over 500 fold less than that of Cdc42GAP  [ **ARHGAP1** ] , respectively, and the differences are due, to a large part, to differences in Km.  Ref: 9268338 J Biol Chem, 1997
- We show here that BNIP 2 and Cdc42GAP  [ **ARHGAP1** ]  could directly bind to each other and they also compete for the binding to the same target,  **Cdc42** .  Ref: 10551883 J Biol Chem, 1999
- In the complex Cdc42Hs  [ **CDC42** ]  interacts, mainly through its switch I and II regions, with a shallow pocket on rhoGAP  [ **ARHGAP1** ]  which is lined with conserved residues.  Ref: 9262406 Nature, 1997
- Proteins containing a RhoGAP  [ **ARHGAP1** ]  Rho GTPase activating protein domain usually function to catalyze the hydrolysis of GTP that is bound to Rho, Rac and or  **Cdc42** , inactivating these regulators of the actin cytoskeleton.  Ref: 12297274 FEBS Lett, 2002

**Non-interactive relationship** (e.g. studied together, co-existance, homology, etc.)

- Our data indicate that the insert region of Cdc42Hs  [ **CDC42** ]  is not essential for its interactions with various target effector molecules or for interactions with the guanine nucleotide exchange factor, Dbl, or the  **Cdc42**  GTPase activating protein  [ **ARHGAP1** ]  GAP.  Ref: 9334181 J Biol Chem, 1997
- In the study presented here, we have tested this idea by examining three residues within the Cdc42GAP  [ **ARHGAP1** ] , which are missing in the GAP homology domain of the 85 kDa regulatory subunit p85 of the PI 3 kinase and are involved in specific interactions with switch domain residues of  **Cdc42** .  Ref: 12501193 Biochemistry, 2002
- Strikingly, this shift in favor of nonpyramidal morphologies is also inhibited by the expression of dominant negative mutants of  **Cdc42**  and Rac and by RhoGAP  [ **ARHGAP1** ]  p190.  Ref: 9331353 Neuron, 1997
- In all cases, tyrosine phosphorylation of BNIP 2 severely impaired its association with Cdc42GAP  [ **ARHGAP1** ]  and its induced GTPase activating protein like activity toward  **Cdc42** .  Ref: 10551883 J Biol Chem, 1999
- In contrast, the BCH domain of Cdc42GAP  [ **ARHGAP1** ] , although it can bind  **Cdc42** , is catalytically inactive.  Ref: 10954711 J Biol Chem, 2000
- BNIP 2 shares a region of homology with the noncatalytic domain of Cdc42GAP  [ **ARHGAP1** ] , a GTPase activating protein for the small GTP binding molecule,  **Cdc42** .  Ref: 10551883 J Biol Chem, 1999
- Moreover, similar to the case of  **Cdc42**  and Cdc42GAP  [ **ARHGAP1** ]  interaction,  **Cdc42**  GDP interacted with tetrafluoroaluminate and  **Cdc42**  GTPgammaS guanosine 5 3 O thio triphosphate to form a transition state complex of the GTPase activating reaction in which the carboxyl terminal determinant s of the GTPgammaS bound  **Cdc42**  plays a critical role.  Ref: 9748241 J Biol Chem, 1998
- Ab initio methods were used to shed light on fundamental aspects of the enzymatic mechanism of guanosine triphosphate hydrolysis in the  **Cdc42**  Cdc42GAP  [ **ARHGAP1** ]  complex.  Ref: 11929266 J Am Chem Soc, 2002
- In contrast with  **Cdc42**  for which the GAP domain of p50RhoGAP  [ **ARHGAP1** ]  is 50 fold more efficient than those of p190, Bcr, and 3BP 1 and with RhoA toward which only p50RhoGAP  [ **ARHGAP1** ]  and p190 displayed high efficiencies, the catalytic efficiencies Kcat Km of the GAP domains of p50RhoGAP  [ **ARHGAP1** ] , p190, Bcr, and 3BP 1 on Rac1 are found to be comparable in a range between 0.9 and 2.6 min 1 microM 1.  Ref: 9535855 J Biol Chem, 1998
- On the basis of the structural determinations of the  **Cdc42**  Cdc42GAP  [ **ARHGAP1** ]  complex, as well as the Ras RasGAP complex, it has been proposed that an arginine residue provided by the GAP called the arginine finger stabilizes charges developing on the guanine nucleotide during the transition state for GTP hydrolysis and is an important contributor to GAP stimulated catalysis.  Ref: 12501193 Biochemistry, 2002
- There is a rotation of 20 degrees between the Rho and rhoGAP  [ **ARHGAP1** ]  proteins in this complex when compared with the ground state complex Cdc42Hs  [ **CDC42** ] .GMPPNP rhoGAP  [ **ARHGAP1** ] , in which Cdc42Hs  [ **CDC42** ]  is bound to the non hydrolysable GTP analogue GMPPNP.  Ref: 9338791 Nature, 1997
- These results suggest that p50RhoGAP  [ **ARHGAP1** ] , p190, Bcr, and 3BP 1 are all capable of acting as a negative regulator for Rac1 mediated signaling, and that, although PAK1 and IQGAP1 can couple tightly with both Rac1 and  **Cdc42** , PAK2 is likely to be a specific effector for Rac1 instead of  **Cdc42** .  Ref: 9535855 J Biol Chem, 1998
- In vitro assays demonstrated that the RhoGAP  [ **ARHGAP1** ]  domain of RICH 1 catalyzed GTP hydrolysis on  **Cdc42**  and Rac1, but not on RhoA.  Ref: 11431473 J Biol Chem, 2001
- Tyrosine phosphorylation of the Bcl 2 associated protein BNIP 2 by fibroblast growth factor receptor 1 prevents its binding to Cdc42GAP  [ **ARHGAP1** ]  and  **Cdc42** .  Ref: 10551883 J Biol Chem, 1999
- We report here the crystal structure of Cdc42Hs  [ **CDC42** ] , with the non hydrolysable GTP analogue GMPPNP, in complex with the GAP domain of p50rhoGAP  [ **ARHGAP1** ]  at 2.7A resolution.  Ref: 9262406 Nature, 1997
- Regulation of  **CDC42**  GTPase by proline rich tyrosine kinase 2 interacting with PSGAP, a novel pleckstrin homology and Src homology 3 domain containing rhoGAP  [ **ARHGAP1** ]  protein.  Ref: 11238453 J Cell Biol, 2001
- Ectopic expression of the RhoGAP  [ **ARHGAP1** ]  domain as well as the full length protein interfered with platelet derived growth factor BB induced membrane ruffling, but not with serum induced stress fiber formation, further emphasizing the notion that, in vivo, RICH 1 is a GAP for  **Cdc42**  and Rac1.  Ref: 11431473 J Biol Chem, 2001
- Furthermore, the p50RhoGAP  [ **ARHGAP1** ]  specificity for  **Cdc42**  was lost in the absence of Mg 2 cofactor.  Ref: 10843989 J Biol Chem, 2000
- The cycling of  **Cdc42**  between its on GTP bound and off GDP bound states is essential for its stimulation of cell growth and transformation, with an important aspect of this cycle being the regulation of the GTP hydrolytic activity of  **Cdc42**  by its GTPase activating protein Cdc42GAP  [ **ARHGAP1** ] .  Ref: 12501193 Biochemistry, 2002
- Biochemical studies of the mechanism of action of the  **Cdc42**  GTPase activating protein  [ **ARHGAP1** ] .  Ref: 9632678 J Biol Chem, 1998
- While ExoT RhoGAP  [ **ARHGAP1** ]  stimulates actin reorganization through the inactivation of Rho, Rac and  **Cdc42** , the function of the ADP ribosylation domain is unknown.  Ref: 12807879 J Biol Chem< MedlineTA>J Biol Chem, 1998
- In this report, we present the tertiary structure for the aluminum fluoride promoted complex between  **Cdc42**  and a catalytically active domain of Cdc42GAP  [ **ARHGAP1** ]  as well as the complex between  **Cdc42**  and the catalytically compromised Cdc42GAP  [ **ARHGAP1** ]  R305A mutant.  Ref: 9846874 Nat Struct Biol, 1998
- Expression of dominant negative mutants of Rac or  **Cdc42** , the Rho inhibitory molecule C3 transferase, or the GTPase activating protein RhoGAP  [ **ARHGAP1** ]  p190 causes a marked reduction in the number of primary dendrites in nonpyramidal multipolar neurons and in the number of basal dendrites in neurons with pyramidal morphologies.  Ref: 9331353 Neuron, 1997
